# Quinacrine inhibits GSTA1 activity and induces apoptosis through G_1_/S arrest and generation of ROS in human non-small cell lung cancer cell lines

**DOI:** 10.18632/oncotarget.27558

**Published:** 2020-05-05

**Authors:** Makhan Kumar, Ansie Martin, Snehal Nirgude, Bibha Chaudhary, Sukanta Mondal, Angshuman Sarkar

**Affiliations:** ^1^CMBL, Department of Biological Sciences, CMBL, BITS Pilani K K Birla Goa Campus, Zuarinagar, Goa 40372, India; ^2^Institute of Bioinformatics and Applied Biotechnology (IBAB), Bangalore, Electronics City Phase 1, Bengaluru, Karnataka 560100, India; ^3^Manipal Academy of Higher Education, Manipal, Karnataka 576104, India; ^4^Present Address: UMR 1236, Faculty of Medicine, Rennes 35043, France

**Keywords:** quinacrine, NSCLC, RhoGTPases, apoptosis, cell cycle

## Abstract

Background: Quinacrine (QC) is popular for its anti-malarial activity. It has been reported exhibiting anti-cancerous properties by suppressing nuclear factor-κB and activating p53 signaling; however, its effect on cellular pathways in human non-small cell lung cancer (NSCLC) has not been studied.

Materials and Methods: Binding of QC with GSTA1 was studied computationally as well as through GST activity assay kit. Cell viability, cell cycle and mitochondrial membrane potential activity were studied using flow cytometry. RT-PCR and western blot were carried out to understand the involvement of various genes at their mRNA as well as protein level.

Results: QC inhibited the activity of GSTA1 approximately by 40–45% which inhibits cell survival and promotes apoptosis. QC reduced viability of NSCLC cells in a dose-dependent manner. It also causes nuclear fragmentation, G_1_/S arrest of cell cycle and ROS generation; which along with disruption of mitochondrial membrane potential activity leads to apoptotic fate.

Conclusions: Results revealed, QC has promising anti-cancer potential against NSCLC cells *via* inhibition of GSTA1, induction of G_1_/S arrest and ROS mediated apoptotic signaling.

## INTRODUCTION

Lung cancer is one of the leading contributors of cancer related mortalities worldwide [1]. Out of all the lung cancer cases Non-small cell lung cancer (NSCLC) accounts for approximately 80% of overall cases diagnosed. NSCLC is sub-categorized into carcinoma, adenocarcinoma and squamous cell carcinoma. Adenocarcinoma and squamous cell carcinoma accounts for most of the NSCLC cases diagnosed. Various well established cell lines representing NSCLC such as A549 and NCI H520 are widely used in carrying out *in vitro* studies. These two cell lines represent Adenocarcinoma (A549) and Squamous cell carcinoma (NCI H520) categories of NSCLC and bear a major difference in the p53 status with A549 being wild type and NCI H520 being mutated at position 146 in DNA binding domain of the protein [2, 3].

Worldwide a lot of emphasis has been given on discovering bioactive compounds which have potential effects on cancer progression, metastatic spread as well as overcoming the chemo resistant adaptation by cancer cells. Quinacrine (QC) is one such synthetic bioactive compound belonging to 9-aminoacridine family of drugs. QC is popularly known as anti-malarial drug and also has been used for treatment of Giardiasis, helminthic infections [4–6], and as a contraceptive medicine for women during 1980’s as well [7, 8]. Quinacrine is internalized into the cells through Vacoular-ATPases (V-ATPases) transport pumps and readily taken with concentrations as less as 25 nM in 30 minutes to 2–3 hour duration [9–11].

There have been few reports of uncovering the anti-cancerous potential of this molecule (QC) on breast, head and neck cancer, gastric and colon cancer cell lines [12–16]. Most of the reported studies have explored and elucidated the anti-cancer activity of QC through suppressing NF-κB and activating p53 signaling pathway which leads to apoptosis. It also has been reported to affect other intracellular molecules when it is internalized and metabolized into the cell [17]. The polypharmacological nature of QC on the cancer associated cellular processes such as proliferation, cell cycle progression, migration and acquiring chemo resistance etc. is not yet properly understood. QC’s effects on lung cancer cells along with the molecular mechanisms have not been reported till date which are among the most lethal and resistant types of cancer.

Two of the major challenges that treatment landscape of NSCLC facing is chemo resistance and metastasis. NSCLC amongst all other types are much more prone to acquire resistance despite the variety and combination of drugs being used. Statistical data available shows worrying figures of resistance acquired in percentage population of patients across spectrum of drugs that are commonly used for the treatment of same [18, 19]. Almost all patients who receive treatment acquire resistance after cycles of treatment given to them. NSCLC cells adapt to the chemotherapeutics through altering numerous cellular pathways such as multidrug efflux pumps (P-glycoprotein, MRP1) [20], inactivating drugs through enhanced activity of enzymes such as GlutathioneS-transferases, metallothioneins (MTs) [21], altering various signaling cascades such as NOTCH, MCAM etc [22, 23]. and many yet to be discovered. GSTA1 gene which encodes for GSTα protein has been linked to various aspects of cancer namely, proliferation, metastasis and drug resistance. GSTA1 is most abundantly expressed in liver, kidney and small intestine. However, it is also abundantly present in lung along with GSTP [24]. It is known to be overexpressed in lung cancer tumors [25, 26] and they mediate multiple cancer associated phenomenon such as promoting nicotine induced metastasis [27], protecting cancer cells from chemotherapeutic induced apoptosis [28], acquiring chemo resistance by inactivating drugs through GSH conjugation and induction of efflux transporters [29]. Multiple inhibitors of GST class proteins have been found and created which inhibits the activity of most of the GST enzymes, but till date only few compounds have shown to exhibit specific inhibition against GSTA1 which amongst all GSTs have been linked most to cancer progression.

Discovery of specific inhibitors and development of new age conjugated drug molecules which can overcome resistance are current challenge and requisites for the treatment, prevention of relapse and disease free survival of the patients.

In the present study we have discovered novel binding of quinacrine with GSTA1 and inhibiting its catalytic activity. This finding has been accompanied with detailed study of the downstream effects of this molecule and novel interaction on viability of cancer cells, cell cycle progression and apoptotic signaling cascade among two non-small cell lung cancer cell lines namely A549 and NCI H520.

## RESULTS

### Quinacrine binds to GSTA1 (GSTα protein) and inhibits its activity of promoting binding of reduced glutathione to active compounds

Various GST class family member proteins were screened computationally for the binding activity with quinacrine using software iGEMDOCK. GSTA1 gene which encodes for GSTα protein was found to be binding with QC among all GST proteins. GSTA1-GSH bound complex structure file (PDB accession no. 1PKW) was selected for further docking study.

Modeled complex of GSTA1-Ethacrynic acid (EAA) was used as reference for affinity, nature and location of binding of quinacrine (QUN) with GSTA1. Best docking poses were selected for analysis and the iGEMDOCK binding energy values obtained were –89.3 (kcal/mol) for QUN and –53.5 (kcal/mol) for EAA. Further, the detailed analysis of the complexes revealed that quinacrine binds mainly to G-site [30] with residues Tyr9 and Gln54 involved in hydrogen bonding, residues Arg15, Glu104, Asp101(chain B) involved in π-cation/anion interaction, and residues Leu107, Ala216, Phe10, Gln67 involved in hydrophobic interaction.

**Figure 1:**
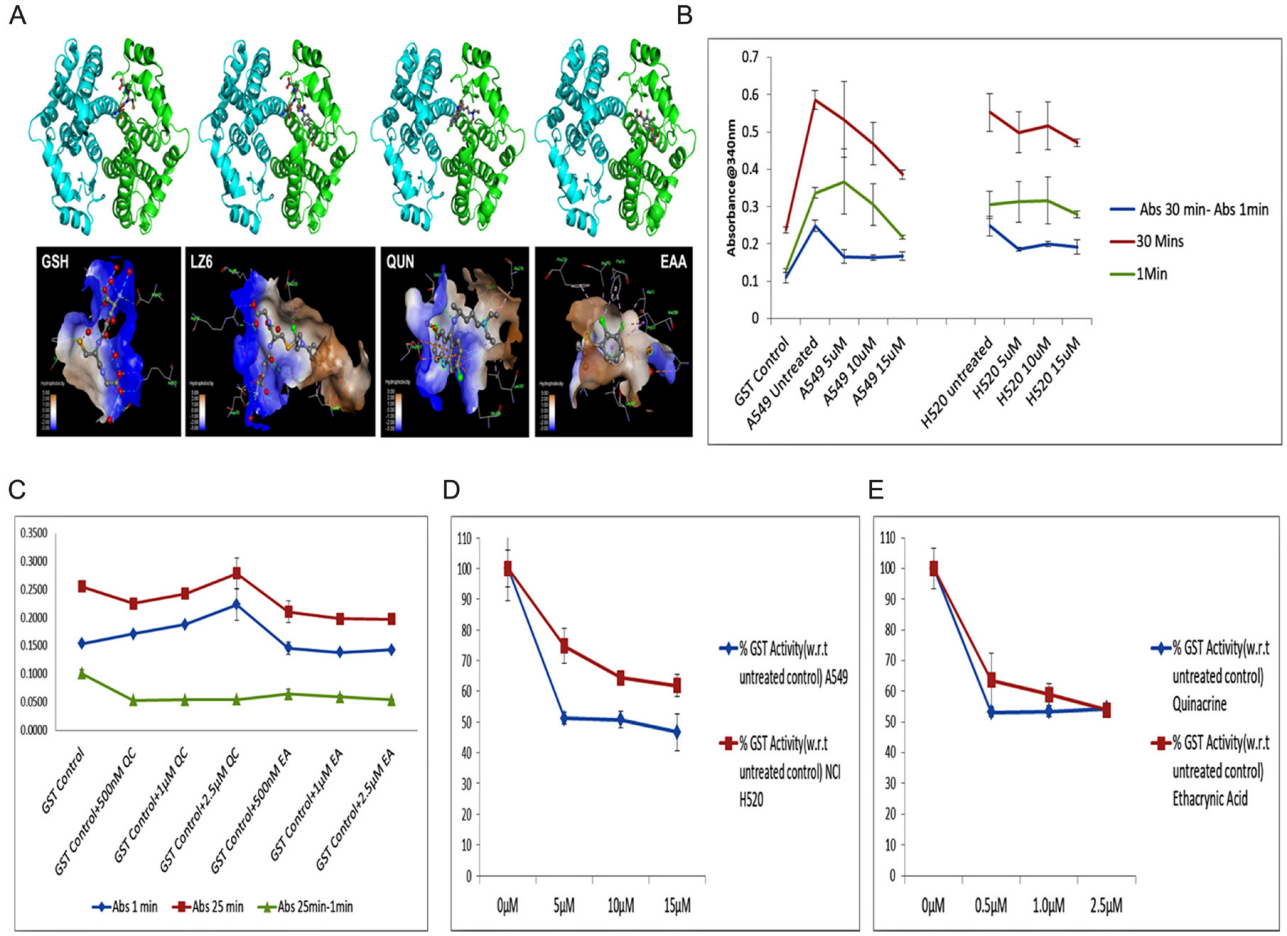
(**A**) Structural representation of binding of (left to right) GSH, LZ6 (Chlorambucil), Quinacrine (QUN) and Ethacrynic acid (EAA) with GSTA1 [Blue ribbon representing A chain, green ribbon representing B chain of GSTA1 and ball and stick representing ligands].Bottom figure displays the ligands in the binding pocket with hydrophobicity and key interacting residues as generated by Discovery studio visualizer (**B**) Graph representing GST assay absorbance values of A549 and NCI H520 QC treated cell lysates (1 min, 30 min and 30-1 min). (**C**) Graph representing GST assay absorbance values of GST Control protein treated/incubated with different concentrations Of QC and EAA (1 min, 25 min and 25-1 min). (**D**) Graph representing the calculated percentage GST activity of cell lysates of A549 and NCI H520 with respect to their untreated controls. (**E**) Graph representing the calculated percentage GST activity of quinacrine and ethacrynic acid treated GST control protein with respect to the untreated sample.

Ethacrynic acid, on the other hand binds mainly to the H-site [30]. Residues Gly14 and Met208 involved in hydrogen bonding, residue Arg13 observed making salt bridges, and residues Phe10, Ala12, Arg15, Leu107, Ala216 and Phe220 involved in hydrophobic interaction (Figure 1A and Table 1). Previous research reports have suggested similar binding site for EAA [31]. To explore whether quinacrine could bind to other isoforms of GST enzymes, we analyzed the locations of key residues of GSTA1 found to be interacting with QUN in various isoforms of GST family proteins through multiple sequence alignment (MSA) of residues of the isoforms (Supplementary Figure 1). It was seen that residues which were found to be interacting with QUN in GSTA1 were not same at similar position in other isoforms at the same location, which would not facilitate the desired interaction of the ligand.

**Table 1:** Table representing the detailed molecular interactions of modeled complexes of QUN and EAA with GSTA1

Bound ligand	Predicted affinity (CSM lig server) (-log_10_ (K_D_|K_i_))	Interaction	Nature of interaction	Distance (Å)
QUN	14.5	[A]Tyr9:OH – QUN:N	Hydrogen bond	3.17
		[A]Gln54:OE1 – QUN:N	Hydrogen bond	3.24
		[A]Arg15:NH1 – QUN	Electrostatic (π-cation)	3.88
		[A]Arg15:NH2 – QUN	Electrostatic (π-cation)	3.44
		[A]Glu104:OE2 – QUN	Electrostatic (π-anion)	3.94
		[B]Asp101:OD1 – QUN	Electrostatic (π-anion)	4.77
		[B]Asp101:OD2 – QUN	Electrostatic (π-anion)	4.21
		[B]Asp101:OD2 – QUN	Electrostatic (π-anion)	4.42
		[A]Phe10 – QUN	Hydrophobic (π-alkyl)	4.40
		[A]Arg15 – QUN	Hydrophobic (alkyl)	4.92
		[A]Gln54 – QUN	Hydrophobic (alkyl)	3.60
		[A]Leu107 – QUN	Hydrophobic (alkyl)	3.95
		[A]Ala216 – QUN	Hydrophobic (alkyl)	4.27
EAA	11.2	[A]Gly14:N – EAA:O	Hydrogen bond	3.78
		[A]Met208:N – EAA:O	Hydrogen bond	4.02
		[A]Arg13:NH1 – EAA:O	Electrostatic (charge-charge)	4.38
		[A]Phe10 – EAA	Hydrophobic (π-alkyl)	4.30
		[A]Phe10 – EAA	Hydrophobic (π-alkyl)	4.12
		[A]Leu107 – EAA	Hydrophobic (π-alkyl)	4.84
		[A]Met208 – EAA	Hydrophobic (π-alkyl)	5.16
		[A]Phe220 – EAA	Hydrophobic (π-alkyl)	5.13
		[A]Ala12 – EAA	Hydrophobic (alkyl)	4.02
		[A]Arg15 – EAA	Hydrophobic (alkyl)	4.58
		[A]Met208 – EAA	Hydrophobic (alkyl)	4.20
		[A]Ala216 – EAA	Hydrophobic (alkyl)	3.85

Modeled molecular interactions of GSTA1 with QUN and EAA.

This finding was experimentally tested with GST activity assay kit using QC treated cell lysates of A549 and NCI H520 and GST control protein provided with kit itself (Liver GST) was used as well for analyzing the effect of QC in comparison with that of ‘Ethacrynic Acid’, which is a well-known inhibitor of GST family of proteins and also used as reference in our docking studies. The GST activity was found to be reduced to 51.17% in 5 μM and to 46.64% in 15 μM QC treated samples of A549 cells compared to untreated control whose activity was considered to be 100%. In NCI H520 cells, the reduction in activity was found to be 74% in 5 μM treated cells which decreased further to 61.91% in 15 μM treated cells compared to untreated control. This variance in inhibitory effect could be due to differential expression of GSTα protein in both cell lines.

To further confirm the inhibition, GST control protein was incubated with different concentrations of QC and E. A as mentioned in the methods section. The results showed 46.78% inhibition of GST activity in 500 nM QC treatment and was found sustaining in higher concentration exposures compared to untreated control sample, whereas, EAA threated samples showed 36.4% inhibition activity in 500 nM treatment which increased further to 46% in 2.5 μM treatment concentration. This result confirmed inhibitory activity of QC on GST (most likely GSTA1) which is comparable with the effects of Ethacrynic acid. (Figure 1B–1E, Table 2).

**Table 2:** Table representing the calculated GST activity of QC treated cell lysates and GST control samples treated with various concentrations of QC and EA

Sl. No	Sample ID	Abs_30 min_@340 nm (Average)	Abs_1 min_@340 nm (Average)	$\frac{\Delta {\text{A}}_{340}/\text{min}={\text{A}}_{340}\left(30\text{ min}\right)-{\text{A}}_{340}\left(1\text{ min}\right)}{30\left(\text{min}\right)-1\;\left(\text{min}\right)}$	$$\begin{array}{lll} \frac{\text{GST Activity}=\Delta {\text{A}}_{340}/\text{min}}{0.00503{\text{ μM}}^{-1}\left(\text{nmol/min}\text{.ml}\right)} & \times & \frac{0.2\text{ ml}\times \text{dil}\text{.f}}{0.02\text{ ml}} \end{array}$$ (Average)	% GST Activity w.r.t to untreated control (%) (Average)
1	GST Control	0.2378	0.1279	0.00378	7.51	–
2	A549 24 h untreated	0.5849	0.3364	0.00856	24.16	100
3	A549 24 h 5 μM QC	0.5333	0.3671	0.00573	12.5	51.17
4	A549 24 h 10 μM QC	0.4681	0.3049	0.00562	12.29	50.82
5	A549 24 h 15 μM QC	0.3856	0.2180	0.00567	11.27	46.64
6	NCI H520 24 h untreated	0.5526	0.3049	0.00854	33.95	100
7	H520 24 h 5 μM QC	0.4982	0.3127	0.00639	25.40	74.81
8	H520 24 h 10 μM QC	0.5159	0.3164	0.00687	21.85	64.35
9	H520 24 h 15 μM QC	0.4711	0.2973	0.00661	21.02	61.91
		Abs_25 min_@340 nm	Abs_0 min_@340 nm	$\frac{\Delta {\text{A}}_{340}\text{/min}={\text{A}}_{340}\left(25\text{ min}\right){\text{-A}}_{340}\left(1\text{ min}\right)}{25\left(\text{min}\right)\text{-1}\left(\text{min}\right)}$		
10	GST Control	0.2552	0.1445	0.00421	8.36	100
11	GST Control+500 nM QC	0.2254	0.1624	0.00224	4.45	53.22
12	GST Control+1 μM QC	0.2424	0.1806	0.00225	4.47	53.46
13	GST Control+2.5 μM QC	0.2785	0.2174	0.00229	4.55	54.42
14	GST Control+500 nM Ethacrynic Acid	0.2104	0.1356	0.00268	5.32	63.63
15	GST Control+1 μM Ethacrynic Acid	0.1982	0.1325	0.00248	4.93	58.97
16	GST Control+2.5 μM EA	0.1977	0.1365	0.00227	4.51	53.94

Assay was performed in triplicates and the values shown are average of three. “Dil.f ” used in GST activity formula means dilution factor.

### Effect of quinacrine on viability of NSCLC cell lines

**Figure 2:**
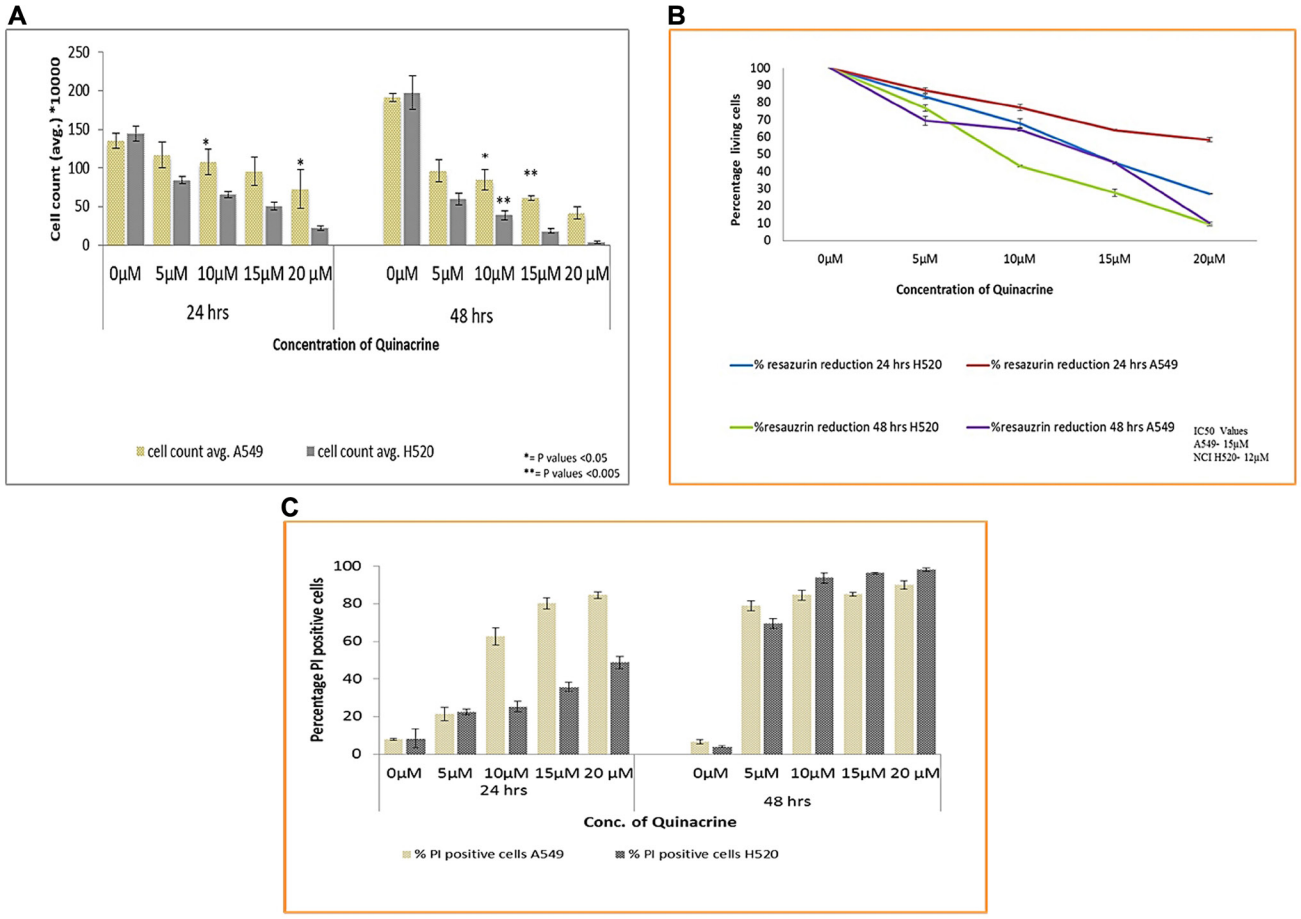
(**A**) Cell viability analysis of A549 and NCI H520 cells after exposure to QC by trypan blue dye exclusion method. Cells were seeded in 6-well plate and treated with various concentrations of QC as described in materials and methods section for 24 and 48 hrs. After that cells were collected and mixed with trypan blue dye in 1:1 ratio and counted under light microscope on Nauber’s hemocytometer. (^*^ = *P* values > 0.05, ^**^ = *P* values > 0.005) (**B**) Anchorage dependent cell viability analysis by resazurin reduction method. Both (A) and (B) represent the mean (± SD) of three independent experiments. (**C**) Graphical representation of Cell viability analysis of both A549 and NCI H520 cells by propidium iodide staining. 1 × 10^5^ cells were seeded in 6-well plate and exposed to various concentrations of QC. Thereafter, cells were collected, washed with PBS and incubated with RNAase A; following that PI stain was added and analysis was done by flow cytometry. Data represent the mean (± SD) of triplicate determinations.

To test whether quinacrine (QC) has an effect on the viability and growth inhibition of Non-small cell lung cancer cell lines, we exposed both A549 and NCI H520 cells to QC in concentrations ranging from 5 to 20 μM for 24 and 48 hrs time points. QC showed a dose and time dependent reduction in the viability of both the cell lines in all three methods namely trypan blue exclusion assay, resazurin reduction assay and viability analysis using Propidium Iodide (PI) dye by flow cytometry. The effect is further enhanced with the prolonged exposure to the molecule as seen In the 48 hrs. time interval cell count by trypan blue dye exclusion method (Figure 2A). The IC_50_ values were calculated using the percentage reduction of resazurin and was found to be 15 μM for A549 cells and 12 μM for NCI H520 cells (Figure 2B). Viability analysis based on PI staining of A549 cells showed an increased percentage of PI positive cells particularly in higher concentrations (15 and 20 μM). Though this followed the trend with other two analysis data in 48 hrs exposure time (Figure 2C and Supplementary Figure 2).

### Quinacrine inhibits cell cycle progression by arresting cells at G_1_-S phase checkpoint

**Figure 3:**
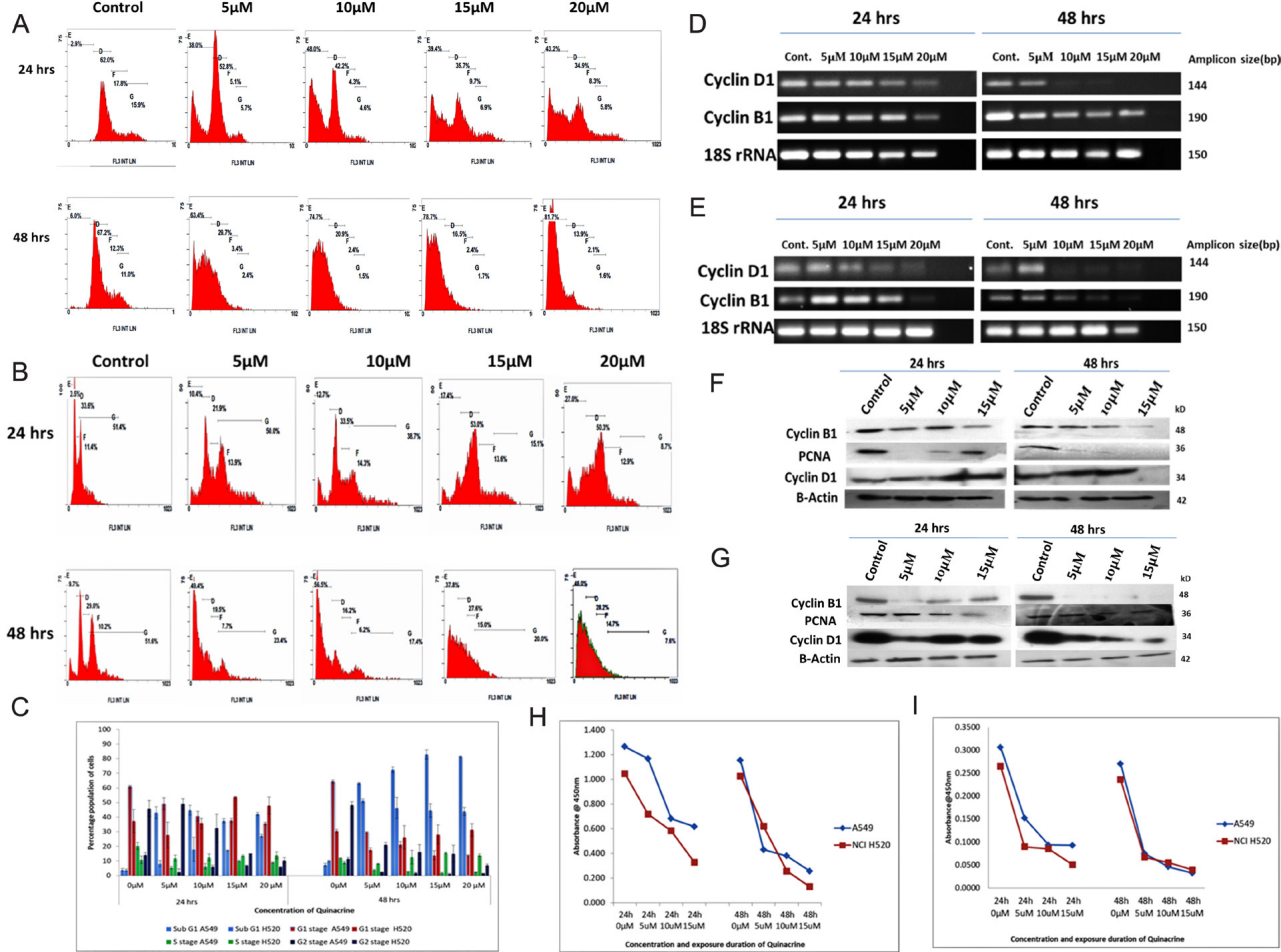
(**A** and **B**) Panel showing Cell cycle profiles of QC exposed A549 and NCI H520 cells respectively analyzed by flow cytometry after staining with Propidium iodide (**C**) Graphical representation of the percentage population of cell cycle stages of QC exposed A549 and NCI H520 cells (light blue represents sub G_1_ stage populations, red, green and navy blue colour representing G_0_/G_1_ stage, S-phase and G_2_/M phase populations respectively). The data shown are the mean (± SD) of triplicate determinations. (**D**) Analysis of QC’s effect on mRNA level expression of Cyclin genes for 24 and 48 hrs time points by RT-PCR in A549 cells. (**E**) Analysis of QC’s effect on mRNA level expression of Cyclin genes for 24 and 48 hrs time points by RT-PCR in NCI H520 cells. (**F**) Protein level expression analysis of Cyclins D_1_ and B_1_ after QC exposure for 24 and 48 hrs by western blot in A549 cells. (**G**) Protein level expression analysis of Cyclins D_1_ and B_1_ after QC exposure for 24 and 48 hrs by western blot in NCI H520 cells. (**H**) Graph representing ELSIA assay of Cyclin B1 protein. (**I**) Graph representing ELSIA assay of PCNA protein.

Quinacrine has a dose dependent effect on the cell cycle progression. In A549 cells the area of peak representing hypodiploid (apoptotic, sub-G_1_/G_0_) population increased to 43% in 20 μM exposure as compared to 2.9% in untreated control cells. The percentage of G_1_/G_0_ population was found to be decreased from 62% in untreated cells to 34.9% in 20 μM exposure concentration. A decrease in G_2_ phase population was also observed from a value of 15.9% in untreated cells to 5.8% in 20 μM concentration, which points out to partial shutdown of the mitotic progression and cell death largely due to nuclear fragmentation (Figure 3A and 3C). NCI H520 cells however, showed a different trend of cell cycle disruption. The lower concentration exposures of QC produced a similar trend to A549 cells with a slight decrease in G_2_ population from 51.8% in untreated cells to 38.7% in 10 μM treated sample in 24 hrs time point. The higher concentration exposure 15 and 20 μM showed a trend which is totally different from the lower concentration exposed pattern. A slight increase in hypodiploid population peak area was observed from a value of 12.7% in 10 μM exposed cells to 27% in 20 μM QC exposed cells. On the contrary, the peak area representing G_1_ population dramatically increased from 21.9% in 5 μM exposed samples to 50.3% in 20 μM exposed samples. That was followed by sharp decline in G_2_ phase population from 38.7% in 10 μM samples to a value of 8.7% in 20 μM exposed samples in 24 hrs. exposure time point itself. Similar trend was observed in 48 hrs.’ time point as well (Figure 3B and 3C).

The mRNA and protein level expression of cell cycle driving protein cyclin D1 was found decreasing dose dependently in both 24 and 48 hrs exposure intervals; whereas the mRNA and protein level expression of Cyclin B1 followed the dose dependent reduction trend similar to cyclin D1 in 24 hrs duration, but the protein expression of cyclin B1 sharply reduced upon the exposure of both lower as well as higher concentrations of quinacrine in 48 hrs exposure duration (Figure 3D–3G, Supplementary Figures 3 and 4). The protein levels of Cyclin B1 and PCNA calculated through ELISA assay was also found following the decreasing trend. Cyclin B1 levels in both A549 and NCI H520 cells decreased nearly 30–40% in 15 μM QC treatment compared to untreated control, which was found further decreased to 50% in 48 hrs exposure duration. The protein levels of PCNA which is a key marker of S-phase progression, was found to be decreased nearly 40–50% in 24 hrs exposure itself, which further dropped down to 60–65% in 48 hours exposure (Figure 3H and 3I) and Table 3. All these results cumulatively provide confirmative proof of dose dependent G_1_-S arrest of cell cycle progression of NSCLC cells.

**Table 3:** Table representing the quantity of Cyclin B1 and PCNA protein in samples calculated through their respective reference standard graph equations

Sl.No	Sample ID	Cyclin B1 protein quantity (ng/ml) (y = 0.4216x –0.8369)	PCNA protein quantity (ng/ml) (y = 0.0533x –0.1099)
1	A549 24 h untreated	4.98	7.81
2	A549 24 h 5 μM QC	4.75	4.92
3	A549 24 h 10 μM QC	3.60	3.81
4	A549 24 h 15 μM QC	3.45	3.79
5	A549 48 h untreated	4.72	7.13
6	A549 48 h 5 μM QC	3.00	3.47
7	A549 48 h 10 μM QC	2.88	2.91
8	A549 48 h 15 μM QC	2.59	2.66
9	NCI H520 24 h untreated	4.46	7.03
10	H520 24 h 5 μM QC	3.69	3.74
11	H520 24 h 10 μM QC	3.37	3.66
12	H520 24 h 15 μM QC	2.76	3.0
13	NCI H520 48 h untreated	4.42	6.48
14	H520 48 h 5 μM QC	3.45	3.31
15	H520 48 h 10 μM QC	2.59	3.09
16	H520 48 h 15 μM QC	2.29	2.79

### Quinacrine causes generation of reactive oxygen species (ROS) leading to ER stress and mitochondria mediated cell death driven by stress activated kinases

**Figure 4:**
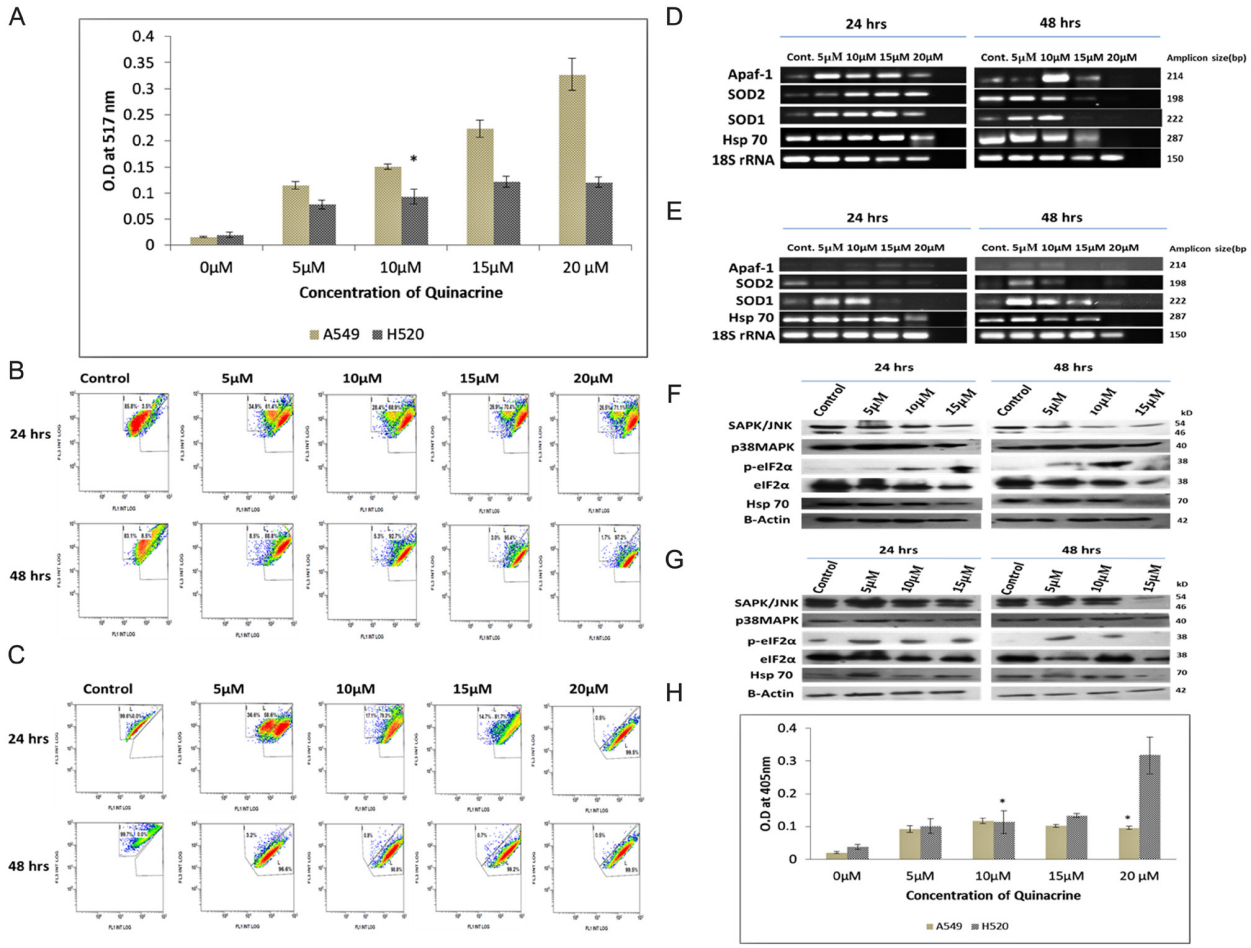
(**A**) Graphical representation of the estimation of reactive oxygen species (ROS) generated due to QC exposure. Data represented here is the mean (± SD) of three independent experiments (^*^ = *P* values > 0.05). (**B**) and (**C**) Analysis of QC’s effect on mitochondrial membrane potential of A549 and NCI H520 cells respectively by JC-1 dye. Cells were grown on 6-well plates and exposed to QC. Post exposure the cells were stained with JC-1 dye and analyzed by flow cytometry. (**D**) Analysis of QC’s effect on mRNA level expression of oxidative stress responsive genes for 24 and 48 hrs time points by RT-PCR in A549 cells. (**E**) Analysis of QC’s effect on mRNA level expression of oxidative stress responsive genes for 24 and 48 hrs time points by RT-PCR in NCI H520 cells. (**F**) Protein level expression analysis of stress kinases and chaperones after QC exposure for 24 and 48 hrs by western blot in A549 cells. (**G**) Protein level expression analysis of stress kinases and chaperones after QC exposure for 24 and 48 hrs by western blot in NCI H520 cells. (**H**) Graphical representation of the concentration of activated caspase-3 protein in A549 and NCI H520 cell lines after QC exposure for 24 hrs time period. Data represented here is the mean (± SD) of three independent experiments (^*^ = *P* values > 0.05).

Quinacrine induces the formation of reactive oxygen species (ROS) in dose dependent manner in lung cancer cells. In A549 cells the ROS generation increased exponentially with increasing concentration of QC, whereas, in NCI H520 cells peak intensity was observed at 15 μM concentration. The peak intensity of ROS generated in NCI H520 cells was found to be roughly three folds lesser as compared to that in A549 cells (Figure 4A).

The activity of the mitochondrial membrane potential was investigated by flow cytometry using JC-1 dye which has dual fluorescence property based on its localization outside or inside of the mitochondrial membrane (Figure 4B and 4C). The assay results showed that A549 cells displayed lesser damage to mitochondrial membrane which increased gradually from approximately 60% on 5 μM exposure to 70% on 20 μM exposure in 24 hrs time period and formation of two distinct population of cells were clearly visible up to 15 μM concentration exposures. On the other hand, NCI H520 cells displayed greater damaged population with increasing concentrations of QC which increased from approximately 58% in 5 μM exposure to approximately 93% in 20 μM exposure in 24 hrs time period. It is interesting to note that the formations of two distinct populations of cells were only observed in 5 μM exposure concentration.

Further, the mRNA expression pattern of superoxide dismutases (SOD), Hsp70 and Apaf-1 gene were also studied which generally are upregulated in cellular response to oxidative stress. The expression of SOD1, SOD2 and Hsp70 were found increased in a dose dependent manner in A549 cells in 24 hrs exposure time, which was followed by sharp reduction in 15 and 20 μM exposures in 48 hrs time point. In case of NCI H520 cells, only the expression of SOD1 gene was found to be increased in 5 and 10 μM exposures and reduced upon subsequent exposure concentrations; whereas the expression of SOD2 gene didn’t increased even upon 5 μM concentration. Hsp70 expression followed the trend similar to A549 cells. The Expression of Apaf-1 increased dose dependently in H520 cells but was found to be decreasing in 15 and 20 μM concentrations in case of A549 cells (Figure 4D and 4E, Supplementary Figures 3 and 6).

The expression of oxidative stress activated proteins were examined by western blot and it was observed that the expression of p38MAPK increased while the expression of total SAPK/JNK decreased dose dependently in A549 cells, whereas, the expression of both proteins were found to be increased up to two folds in NCI H520 cells. Expression of Hsp70 increased up to two-fold in 5 and 10 μM exposures and decreased in subsequent exposure concentrations in case of A549 cells. Though in case of NCI H520 cells it marginally increased in 5 μM concentration and thereby decreased in subsequent concentrations. Expression of phosphorylated eIF2α was found to be marginally increased in dose dependent manner in A549 cells, and increased sharply up to four-folds in NCI H520 cells in 5 μM concentration and remained alleviated in higher concentration exposures (Figure 4F and 4G, Supplementary Figure 4).

To ascertain whether the nature of cell death is majorly caspase dependent or independent, activity of Caspase 3 was examined for 24 hrs. exposure time period which revealed that caspase 3 levels were increased up to 10 μM and reduced in subsequent higher concentrations in A549 cells. Whereas, in case of NCI H520 cells, it increased in a dose dependent manner; and was found to be more than three-fold enhanced at 20 μM concentration exposure as compared to the similar exposure concentration in A549 cells (Figure 4H).

## DISCUSSION

Non-small cell lung cancer till date remains one of the most lethal malignancies which have shown to be more resistant and adaptive to even advanced medications available. The current standard therapeutics used for treatment of NSCLC is also heavily cytotoxic to normal cells and more importantly these cancer cells have been seen to acquire resistance to therapies by various counter-molecular mechanisms. Quinacrine is perhaps one of the most popularly used synthetic bioactive molecule which has clinically been proven to be far less toxic compared to modern day chemotherapeutics and its side effects are extensively studied and well cured. Cancer cells harbor various mutations including that of p53 gene which allows proliferating extensively. Along with discovering new novel molecular targets which could play key roles in cancer suppression, it is, therefore, essential to develop an understanding about the QC’s mechanism of action in p53 mutated cancer cells in comparison with wild type p53 bearing cancer cells; for better assessment of its chemotherapeutic potential.

Inhibition of GSTA1 by quinacrine is novel finding which reveals a new molecular mechanism of QC’s activity. It has been seen that quinacrine binds to the G-site which overlaps to the binding site of GSH to some extent. The predicted binding affinity of quinacrine was higher in comparison to ethacrynic acid. Quinacrine was also seen to be interacting with Tyrosine 9(Tyr9) through hydrogen bonding, which is important for the catalytic activity of GSTA1.

Our experimental results have also concluded the inhibition activity of 49–54% in treated A549 cell lysates and 25–38% in treated NCI H520 cell lysates (5–15 μM treatment). Similar range of inhibition was seen in treated control protein which also is at par with the inhibition activity of Ethacrynic acid tested along with. These results in combination conclude the molecular activity inhibition of GSTA1 by quinacrine which can have promising implications in cancer treatment. GSTA1 is known to inactivate alkylating drugs such as Busulfran, Brostallicin, Carboplatin, cyclophosphamides and anthracycline drugs such as doxorubucin etc. through glutathione conjugation [32, 33]. In addition to its catalytic activity, GSTA is also involved in preventing JNK1 induced apoptosis in cases of oxidative stress and cytokine mediated inflammatory response. Downregulation of GSTA1 has been shown to suppress growth and apoptosis induction in lung cancer cells [34]. GST inhibitors are being used in conjugation with chemotherapeutic drugs to overcome the resistance aspect, Ethacraplatin being an example of it. Few antimalarial drugs such as Quinine, Quinidine have been reported to exhibit inhibitory effect on GSTM1, GSTP1 [35]; however, none of them has been reported to inhibit GSTA1 specifically, which has been found overexpressed specially in lung cancer.

Our study has shown that QC effectively inhibits the proliferation of NSCLC cells in a dose and time dependent manner. QC reduced the viability of both the cells lines up to 50% in 24 hrs. exposure time. This indicates NSCLC cells are more resistant as compared to various other types of cancer cells to the activity of quinacrine which have been reported to exhibit upto 80% cell death in same time duration of treatment [14, 15]. The Propidium Iodide staining showed an enhanced positive percentage population in 24 hrs. for A549 cells as compared to trypan blue and resazurin reduction assay results; however, it followed the similar trend as of the other two assays in 48 hrs exposure time point. This might be suggestive of increased intake of quinacrine molecules by A549 cells as compared to that in NCI H520 cells.

Quinacrine inhibited cell cycle progression as well by arresting the cells at G_1_-S checkpoint through increasing fragmented DNA population in A549 cells and terminally arresting cells at G_0/1_ stage in NCI H520 cells. It also downregulated the expression of Cyclins D_1_ and B_1_ at both transcriptional and translational levels along with approximately two fold decline in PCNA protein which is a DNA polymerase associated protein. These effects cumulatively leads to shutdown of the cell cycle machinery [36]. QC’s effect on regulation of cyclin proteins unravels the molecular mechanisms of its inhibitory action on cancer cell proliferation and had provided conclusive proof of inhibition via G_1_-S checkpoint arrest.

Quinacrine is directly responsible for the generation of intracellular ROS leading to oxidative stress in cell and causes devastating effects on cellular processes such as DNA damage (Supplementary Figure 5), endoplasmic reticulum stress etc. These effects trigger the apoptotic process when the damage is beyond the repair capability [37–39]. Our results indicate that ROS is generated in a dose dependent manner, which sustains due to inefficient dissolution by GSTs, Superoxide dismuatses and other ROS responsive genes. This results to activation of stress kinases and shutdown of the global protein synthesis by phosphorylation of eIF2α which acts as one of determining factors of cell fate [40]. Alleviated ROS also caused pronounced damage to the outer membrane of mitochondria leading to formation of transition pores (mPTPs) which releases cytochrome C into cytoplasm and accelerates apoptosome formation [41]. A549 cells though receive damage to mitochondrial membrane; their apoptosome assemblies fail to process procaspase-9, as suggested by many reports [42, 43] and undergoes apoptosis by other pathways independent of caspases including DNA fragmentation with involvement of mitochondria mediated mechanism to some extent. NCI H520 cells, on the other hand, meet the apoptotic fate centrally driven through mitochondria mediated caspase activation in assistance with ER stress and DNA damage.

Inhibition of GSTA1 and ROS generation are two phenomenon’s’ that are closely connected as GST inhibition results to insufficient dissolution of ROS and enhances the cellular damage by oxidative stress. Few other studies have also pointed out the ROS production effect of quinacrine [44, 45], though the mechanism by which it happens is not clearly understood yet. GSTA1 inhibition itself could be a factor in enhancement of ROS due to accumulation of generated nitric oxide, H_2_O_2_ and other super oxides.

NSCLC cells with mutated tumor suppressor genes respond differently to drugs and are known to be resistant and aggressive. Quinacrine has shown a promising potential to overcome such factors by inhibiting key protein GSTα (encoded by GSTA1) and targeting multiple cellular pathways that results to cell cycle arrest, apoptosis as well as overcoming drug resistance (Supplementary Figure 7). The evaluation of these *in vitro* results in animal model will further strengthen and prove its anti-cancer potential which can lead to formation of new quinacrine based drug conjugates for more effective therapy with lesser side effects.

## MATERIALS AND METHODS

### Purchase of media, reagents, chemicals and antibodies

Cell lines A549 and NCI H520 were purchased from ‘National Center for Cell Science’ (NCCS), Pune, India. Cells were tested for mycoplasma contamination and were found negative. Dulbecco’s modified eagle medium (DMEM) high glucose and RPMI 1640 media and Trypan blue 0.4% solution in PBS was purchased from HIMEDIA, India and fetal bovine serum was purchased from Seralabs, UK for the cell culture work. Quinacrine dihydrochloride powder (Q 3251), Resazurin sodium powder, Phenylmethanesulfonyl chloride (PMSF) and Ponceau S, practical grade powder were purchased from Sigma life Sciences. Protease inhibitor cocktail tablets (EDTA-free) were purchased from Roche diagnostics. Tris Base, Glycine, Acrylamide and Bis-acrylamide powder were purchased from MP biomedicals, India and all other routine chemicals were purchased from Fisher scientific India.

Ribozol RNA extraction reagent was purchased from Amresco life sciences, Verso cDNA synthesis kit from Thermo Scientific and the primers used for RT-PCR analysis were designed and checked for specificity using NCBI Primer BLAST and were ordered from IDT.

Primary antibodies for β-Actin, Hsp 70 and cyclin B1 were purchased from Sigma life sciences. Primary antibodies for eIF2α, Phospho-eIF2α, p38MAPK, SAPK/JNK and p-Rac1/cdc42 were purchased from Cell Signaling technology, MA, USA. E and N- cadherin antibodies (67A4 and 8C11 respectively) were purchased from Bio Legend life sciences and antibodies for Cyclin D1 and Vimentin (SC-450 and SC-6260 respectively) were obtained from Santa Cruz biotechnology. HRP linked secondary antibodies (both anti-mouse and anti-rabbit) were purchased from GE healthcare UK limited. ECL substrate was purchased from Thermo Scientific private limited. JC-1 dye and GST activity assay kit was purchased from Cayman chemicals (item no.10009172 and 703302 respectively) and Cyclin B1 and PCNA ELISA kit was purchased from Elabscience pvt. Ltd, USA (catalog no. E-EL-H0293 and E-EL-H2399 respectively).

### Cell culture and preparation of quinacrine solution

Lung adenocarcinoma cell line A549 and squamous cell carcinoma NCI H520 were purchased and were cultured in the laboratory supplemented with 10% fetal Bovine serum. Cells were maintained at 37°C with 5% CO2 and 95% humidity in CO_2_ incubator. Cells were grown for 5–6 passages and checked under microscope every alternate day for healthy state before being used for experimentation purpose. Cells with passage number P34 to P55 of A549 and Passage number P18 to P39 of NCI H520 were used in this study.

Quinacrine Dihydrochloride powder purchased from Sigma (Q 3251) was weighed and dissolved in tissue culture grade sterile water to make 1 mM stock solution and was kept in 4°C under light protected condition. From this stock solution cells were exposed in 5, 10, 15 and 20 μM/ml concentrations for all experiments carried out as mentioned in this article.

### Trypan blue dye exclusion assay

Approximately 5 × 10^4^ cells were seeded in a 6-well plate and grown for 24 hrs. in CO_2_ incubator. After 24 hrs, growth media was replaced with fresh equal volume and cells were exposed to various concentrations of Quinacrine (QC) as mentioned earlier for 24 and 48 hrs.’ time interval. After that cells were collected by trypsinization, centrifuged at 448×g for 5 mins and resuspended in 100 μl of media and mixed with trypan blue dye solution in 1:1 ratio and counted using Nauber’s hemocytometer (Tiefe Depth Profondeur, Marienfeld, Germany) under microscope.

### Resazurin reduction assay

Cytotoxicity of the drug quinacrine was assessed using resazurin (7-hydroxy-10-oxidophenoxazin-10-ium-3-one) dye which is reduced to fluorescent pink resorufin by live cells. Briefly 5 × 10^4^ cells were plated in 24 well plates and exposed to various concentrations of Quinacrine as mentioned before. After 24 and 48 hrs time interval 10 μl of the stock solution of resazurin dye was added to all wells along with a negative control having no cells and incubated for 4 hrs. After incubation the absorbance values at 600 and 690 nm were taken using Shimazdu spectrophotometer and Positive difference in absorbance at wavelength of 600 nm and 690 nm of each well culture against control was assessed and the percentage reduction was then calculated and reported as a measure of toxicity as per published reports [46, 47].

### Cell viability assessment using propidium iodide dye by flow cytometry

1 × 10^5^ cells were plated in 6-well plates in duplicates and were exposed to different concentrations of quinacrine for 24 and 48 hrs. After that cells were collected and centrifuged at 448×g for 10 mins. Collected pellet was washed twice with 1× PBS buffer and incubated with RNAaseA for 15 mins at 37°C for 15 mins. After the incubation 1 μl of 10 mg/ml stock of Propidium iodide solution was added and immediately analyzed using Beckman Coulter Gallios flow cytometer.

### Cell cycle analysis by flow cytometry

1 × 10^5^ cells were plated in 6-well plates in duplicates and were exposed to different concentrations of Quinacrine for 24 and 48 hrs. After that cells were collected and centrifuged at 448×g for 10 mins. The pellet was then re-suspended in ice cold 80% ethanol and kept overnight under refrigerated conditions for cell lysis. After that the tubes were centrifuged at 252×g for 10 mins and washed with PBS twice and re-suspended in 200 μl of PBS. After that tubes were incubated with RNAase A for 15 mins and 15 mins with 1 μl of 10 mg/ml stock of Propidium iodide solution in dry bath at 37°C. Then they were analyzed using Beckman Coulter Gallios flow cytometer.

### Mitochondrial membrane potential activity assay by flow cytometry

Mitochondrial membrane potential activity was analyzed using JC-1 mitochondrial membrane potential activity kit purchased from Cayman chemicals (10009172). The assay was performed as per the manufacturer’s instructions using Beckman Coulter Gallios flow cytometer.

### RNA isolation and RT-PCR analysis

Total RNA was isolated directly from 25 cm^2^ cell culture flask. Briefly cells were grown and exposed to different concentrations of quinacrine in 25 cm^2^ cell culture flasks. The total RNA was isolated using Ribozol reagent and 1 μg of total RNA was reverse transcribed to cDNA using the cDNA synthesis kit as per the manufacturer’s instructions. This first strand was then further used for amplification of selected targets using specific primers (Supplementary Table 1) by RT-PCR method. 18S rRNA was used as internal control and the band densitometry analysis was performed using NIH Image J software.

### Protein extraction and Western blotting

Total cellular protein was extracted using lysis buffer (Tris 10 mM pH7.4, EDTA, 1 mM, pH 7.4, PMSF, protease inhibitor cocktail tablet, Tritron X-100) on ice bath followed by centrifugation at 16,128×g for 20 mins at 4°C. Quantification was done using the Bradford method and approximately 40 μg of cell lysate was run and normalized on 10–12% SDS-PAGE gel. The resolved gel was then transferred on PVDF membrane using wet transfer system (25V and for 2 hrs.). After that the membrane was blocked with 5% Non-fat dry milk/ BSA in Tris Buffered Saline (TBS) for 2–3 hrs. and then incubated overnight with primary antibodies of various targets prepared in the blocking solution. After primary incubation, blots were incubated with HRP conjugated secondary antibody which was followed by detection on Amershan ECL Hyperfilm.

### Hoechst 3342 staining

Cells were seeded in 6-well plates on coverslips and after 24 hrs. were exposed to various concentrations of QC for 24 and 48 hrs. Post exposure cells were washed with 1×PBS and fixed with 4% paraformaldehyde for 5 mins. Following that cells were stained with Hoechst 3342 purchased from Abcam (ab 145597) as per the manufacturer’s instructions.

### Caspase-3 activity assay

Caspase 3 activity assay kit was purchased from BioVision life sciences (K-106) and the assay was performed as per manufacturer’s instructions.

### ROS (reactive oxygen species) estimation assay using H2DCFDA

Cells were seeded in 96-well plate and after 24 hrs. were incubated with 30 μg of H2DCFDA for 2 hrs. Post incubation cells were treated with various concentrations of Quinacrine for 4 hrs and then were washed twice with PBS and O. D values were taken using spectrophotometer at wavelength of 517 nm.

### GST activity assay

Glutathione S-Transferase assay kit was purchased from Cayman chemicals. For analyzing GST activity in QC treated and untreated cell lysates of A549 and NCI H520 cells, 100 μg of cell lysate were taken and diluted in sample dilution buffer to make sample volume of 20 μl as recommended in manufacturer’s instruction manual for performing the assay. For confirmation of the effect of Quinacrine and its comparison to the effect of well-known inhibitor Ethacrynic acid, three different concentrations (500 nM, 1 μM and 2.5 μM) of both compounds were added into separate wells along with reduced glutathione and GST control protein provided with kit and incubated at 25°C for 10 mins prior to adding CDNB. This assay was performed separately. Both the assays were performed in triplicates and as per manufacturer’s instruction and the GST activities of samples were calculated accordingly.

### Cyclin B1 and PCNA ELISA assay

Cyclin B1 and PCNA ELISA assay kits were obtained from Elabscience pvt. Ltd. 40 ug of cell lystaes were taken for the assay and performed as per manufacturer’s instruction.

### Molecular docking study of GSTA1

Computational docking of the quinacrine and Ethacrynic acid onto GSTA1 was performed using iGEMDOCK docking program [48]. Stable docking option with population size *N* = 300, generations (80) and no. of solution (10) was selected. Repeated studies were performed with same compound and settings to avoid false positive and negative results.

Structure files of Glutathione (GSH) in complex with GSTA1 (PDB accession no.1PKW), GSTA1 in complex with ligand Chlorambucil (PDB accession no.4HJ2) were downloaded from RCSB protein databank [49]. Structure of compound Ethacrynic acid (EAA) was taken from bound complex with GSTP (PDB accession no. 2GSS). Residues in proximity of 6–8 Å radius of the ligand (EAA) were selected and structurally equivalent residues in the structure file 1PKW used as binding site for docking study. 3D structure file of Quinacrine (QUN) was also downloaded from RCSB database.

### Post docking analysis

Protein ligand interaction for modeled complexes generated by software Protein ligand interaction profiler (PLIP), PyMOL and Discovery studio visualizer version 19.1 were studied for detailed understanding of the interactions [50–52]. GSM-lig server was used for predicting binding affinities of QUN and EAA [53].

### Statistical analysis

Statistical significance of difference between QC treated and untreated samples were determined by two tailed Student’s *t*-test and *p* values greater than 0.05 were considered statistically significant.

## SUPPLEMENTARY MATERIALS


