# Correction: Quinacrine inhibits GSTA1 activity and induces apoptosis through G_1_/S arrest and generation of ROS in human non-small cell lung cancer cell lines

**DOI:** 10.18632/oncotarget.27821

**Published:** 2020-11-17

**Authors:** Makhan Kumar, Ansie Martin, Snehal Nirgude, Bibha Chaudhary, Sukanta Mondal, Angshuman Sarkar

**Affiliations:** ^1^ CMBL, Department of Biological Sciences, CMBL, BITS Pilani K K Birla Goa Campus, Zuarinagar, Goa 40372, India; ^2^ Institute of Bioinformatics and Applied Biotechnology (IBAB), Bangalore, Electronics City Phase 1, Bengaluru, Karnataka 560100, India; ^3^ Manipal Academy of Higher Education, Manipal, Karnataka 576104, India; ^4^ Present Address: UMR 1236, Faculty of Medicine, Rennes 35043, France

**This article has been corrected:** Due to a naming mistake during the original image capture process, the 24-hour 20 microMolar image in Figure 4C is an accidental duplicate of the 48-hour 20 microMolar image. The corrected Figure 4, obtained using the original data, is shown below. The authors declare that these corrections do not change the results or conclusions of this paper.

Original article: Oncotarget. 2020; 11:1603–1617. 1603-1617. https://doi.org/10.18632/oncotarget.27558

**Figure 4:**
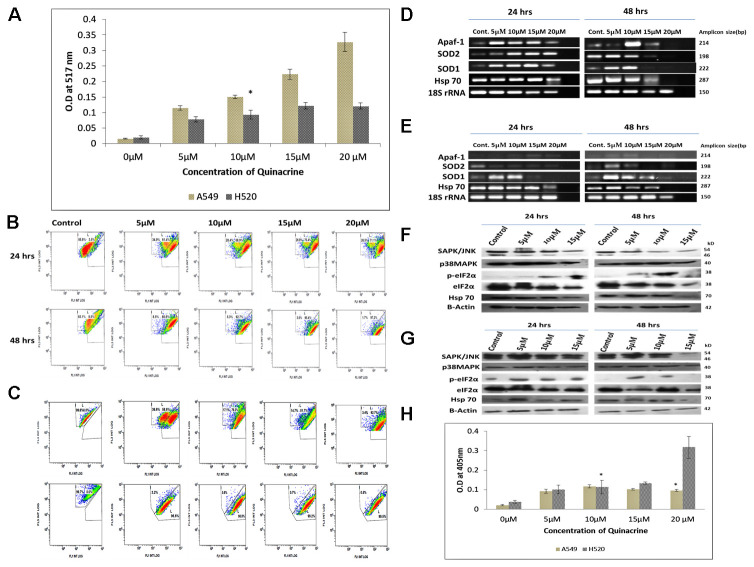
(**A**) Graphical representation of the estimation of reactive oxygen species (ROS) generated due to QC exposure. Data represented here is the mean (± SD) of three independent experiments (^*^ = *P* values > 0.05). (**B**) and (**C**) Analysis of QC’s effect on mitochondrial membrane potential of A549 and NCI H520 cells respectively by JC-1 dye. Cells were grown on 6-well plates and exposed to QC. Post exposure the cells were stained with JC-1 dye and analyzed by flow cytometry. (**D**) Analysis of QC’s effect on mRNA level expression of oxidative stress responsive genes for 24 and 48 hrs time points by RT-PCR in A549 cells. (**E**) Analysis of QC’s effect on mRNA level expression of oxidative stress responsive genes for 24 and 48 hrs time points by RT-PCR in NCI H520 cells. (**F**) Protein level expression analysis of stress kinases and chaperones after QC exposure for 24 and 48 hrs by western blot in A549 cells. (**G**) Protein level expression analysis of stress kinases and chaperones after QC exposure for 24 and 48 hrs by western blot in NCI H520 cells. (**H**) Graphical representation of the concentration of activated caspase-3 protein in A549 and NCI H520 cell lines after QC exposure for 24 hrs time period. Data represented here is the mean (± SD) of three independent experiments (^*^ = *P* values > 0.05).

