# Supplementary material for: Quinacrine inhibits GSTA1 activity and induces apoptosis through G1/S arrest and generation of ROS in human non-small cell lung cancer cell lines
Source: Oncotarget. 2020 May 5;11(18):1603–17. doi: 10.18632/oncotarget.27558 (PMC7210017; doi:10.18632/oncotarget.27558)
Supplement: Supplementary file 1 [file oncotarget-11-1603-s001.pdf]

Quinacrine inhibits GSTA1 activity and induces apoptosis through G_1/S arrest and generation of ROS in human non-small cell lung cancer cell lines

SUPPLEMENTARY MATERIALS

Supplementary Table 1: Table representing the details of the primers used for amplifying target genes by RT-PCR as shown in results

SL. No	Target gene Primer sequence	Product size (bp)
1.	18SrRNA- Forward-5′-GTAACCCGTTGAACCCCATT-3′. Reverse-5′-CCATCCAATCGGTAGTAGCG-3′.	150
2.	Cyclin D1- Forward-5′-ACAAACAGATCATCCGCAAACAC-3′. Reverse-5′-TGTTGGGGCTCCTCAGGTTC-3′.	144
3.	Cyclin B1- Forward-5′-CAGTCAGACCAAAATACCTACTGGGT-3′. Reverse-5′-ACACCAACCAGCTGCAGCATCTTCTT-3′.	190
4.	Hsp70- Forward-5′-CACTCTGCTTATCAAGTTTC-3′. Reverse-5′-GTTCCAGATACATCAACATC-3′.	287
5.	SOD1- Forward-5′-CAGTGCAGGGCATCATCAAT-3′. Reverse-5′-CATTGCCCAAGTCTCCAACA-3′.	222
6.	SOD2- Forward-5′-CGACCTGCCCTACGACTACG-3′. Reverse-5′-TGACCACCACCATTGAACTT-3′.	198
7.	Apaf-1- Forward-5′-CACGTTCAAAGGTGGCTGAT-3′. Reverse-5′-TGGTCAACTGCAAGGACCAT-3′.	214

```
CLUSTAL O(1.2.4) multiple sequence alignment

SP|P08263|GSTA1_HUMAN MAEKPKLHYFNARGRMESTRWLLAAAGVEFEEKFIKSAEDLDKL-------RNDGYLMFQ 53
SP|P09210|GSTA2_HUMAN MAEKPKLHYSNIRGRMESIRWLLAAAGVEFEEKFIKSAEDLDKL-------RNDGYLMFQ 53
SP|P09211|GSTP1_HUMAN -MPPYTVVYFPVRGRCAALRMLLADQGQSWKEEVVT--------VETWQEGSLKASCLYG 51
SP|P09488|GSTM1_HUMAN --MPMILGYWDIRGLAHAIRLLLEYTDSSYEEKKYTMGDAPDYDRSQWLNEKFKLGLDFP 58
SP|P28161|GSTM2_HUMAN --MPMTLGYWNIRGLAHSIRLLLEYTDSSYEEKKYTMGDAPDYDRSQWLNEKFKLGLDFP 58
SP|P46439|GSTM5_HUMAN --MPMTLGYWDIRGLAHAIRLLLEYTDSSYVEKKYTLGDAPDYDRSQWLNEKFKLGLDFP 58
SP|P30711|GSTT1_HUMAN ---MGLELYLDLLSQPCRAVYIFAKKNDIPFELRIV-----DLIKGQHLSDAFAQVNPLK 52
                              *

SP|P08263|GSTA1_HUMAN QVPMVEIDGMKLVQTRAILNYIASKYNL----YGKDIKERALIDMYIEGIADLGEMILLL 109
SP|P09210|GSTA2_HUMAN QVPMVEIDGMKLVQTRAILNYIASKYNL----YGKDIKEKALIDMYIEGIADLGEMILLL 109
SP|P09211|GSTP1_HUMAN QLPKFQDGDLTLYQSNTILRHLGRTLGL----YGKDQQEAALVDMVNDGVEDLRCKYISL 107
SP|P09488|GSTM1_HUMAN NLPYLIDGAHKITQSNAILCYIARKHNL----CGETEEEKIRVDILENQTMDNHMQLGMI 114
SP|P28161|GSTM2_HUMAN NLPYLIDGTHKITQSNAILRYIARKHNL----CGESEKEQIREDILENQFMDSRMQLAKL 114
SP|P46439|GSTM5_HUMAN NLPYLIDGAHKITQSNAILRYIARKHNL----CGETEEEKIRVDILENQVMDNHMELVRL 114
SP|P30711|GSTT1_HUMAN KVPALKDGDFTLTESVAILLYLTRKYKVPDYWYPQDLQARARVDEYLAWQ---HTTLRRS 109
                        *              **                        *

SP|P08263|GSTA1_HUMAN PVCPPEEKDAKLALIKEKIKNRYFPAFEKVLK----------SHGQDYLVGNKLSRADIH 159
SP|P09210|GSTA2_HUMAN PFSQPEEQDAKLALIQEKTKNRYFPAFEKVLK----------SHGQDYLVGNKLSRADIH 159
SP|P09211|GSTP1_HUMAN IY-TNY----------EAGKDDYVKALPGQLK-PFETLLSQNQGGKTFIVGDQISFADYN 155
SP|P09488|GSTM1_HUMAN CYNPEF----------EKLKPKYLEELPEKLK-LYS----EFLGKRPWFAGNKITFVDFL 159
SP|P28161|GSTM2_HUMAN CYDPDF----------EKLKPEYLQALPEMLK-LYS----QFLGKQPWFLGDKITFVDFI 159
SP|P46439|GSTM5_HUMAN CYDPDF----------EKLKPKYLEELPEKLK-LYS----EFLGKRPWFAGDKITFVDFL 159
SP|P30711|GSTT1_HUMAN CLRALWHKVMFPVFLGEPVSPQTLAATLAELDVTLQLLEDKFLQNKAFLTGPHISLADLV 169
                                      *      .      *                   *      *

SP|P08263|GSTA1_HUMAN LVELLYYVEELDSSLISSFPLLKALKTRISNLPTVKKFLQPG---------SPRKPPMDE 210
SP|P09210|GSTA2_HUMAN LVELLYYVEELDSSLISSFPLLKALKTRISNLPTVKKFLQPG---------SPRKPPMDE 210
SP|P09211|GSTP1_HUMAN LLDLLLIHEVLAPGCLDAFPLLSAYVGRLSARPKLKAFLASP---------EYVNLPING 206
SP|P09488|GSTM1_HUMAN VYDVLDLHRIFEPKCLDAFPNLKDFISRFEGLEKISAYMKSS---------RFLPRPVFS 210
SP|P28161|GSTM2_HUMAN AYDVLERNQVFEPSCLDAFPNLKDFISRFEGLEKISAYMKSS---------RFLPRPVFT 210
SP|P46439|GSTM5_HUMAN AYDVLDMKRIFEPKCLDAFLNLKDFISRFEGLKKISAYMKSS---------QFLRGLLFG 210
SP|P30711|GSTT1_HUMAN AITELMHPVGAGCQVFEGRPKLATWRQRVEAAVGEDLFQEAHEVILKAKDFPPADPTIKQ 229
                          *                *     *

SP|P08263|GSTA1_HUMAN KSLEEARKIFRF 222
SP|P09210|GSTA2_HUMAN KSLEESRKIFRF 222
SP|P09211|GSTP1_HUMAN NGKQ-------- 210
SP|P09488|GSTM1_HUMAN KMAVWGNK---- 218
SP|P28161|GSTM2_HUMAN KMAVWGNK---- 218
SP|P46439|GSTM5_HUMAN KSATWNSK---- 218
SP|P30711|GSTT1_HUMAN KLMPWVLAMIR- 240
```

Supplementary Figure 1: Multiple sequence alignment of GST isoforms. Protein sequences were extracted from UniProtKB database (https://www.uniprot.org) and aligned with Clustal Omega program (http://www.clustal.org/omega/). Putative QC interacting residues of GSTA1 are highlighted in red.

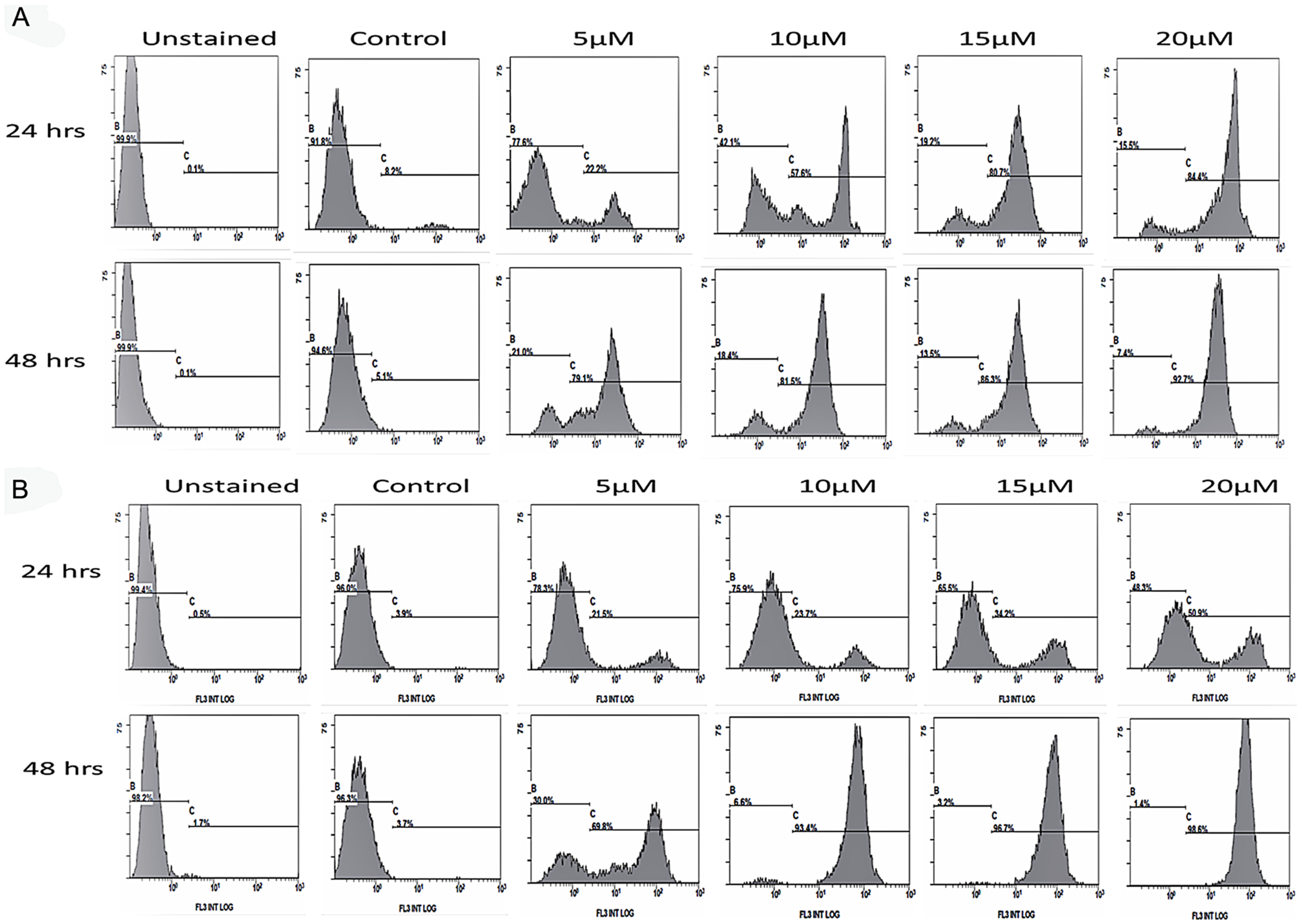

Supplementary Figure 2: Histogram of cell viability analysis by propidium iodide using flow cytometry.

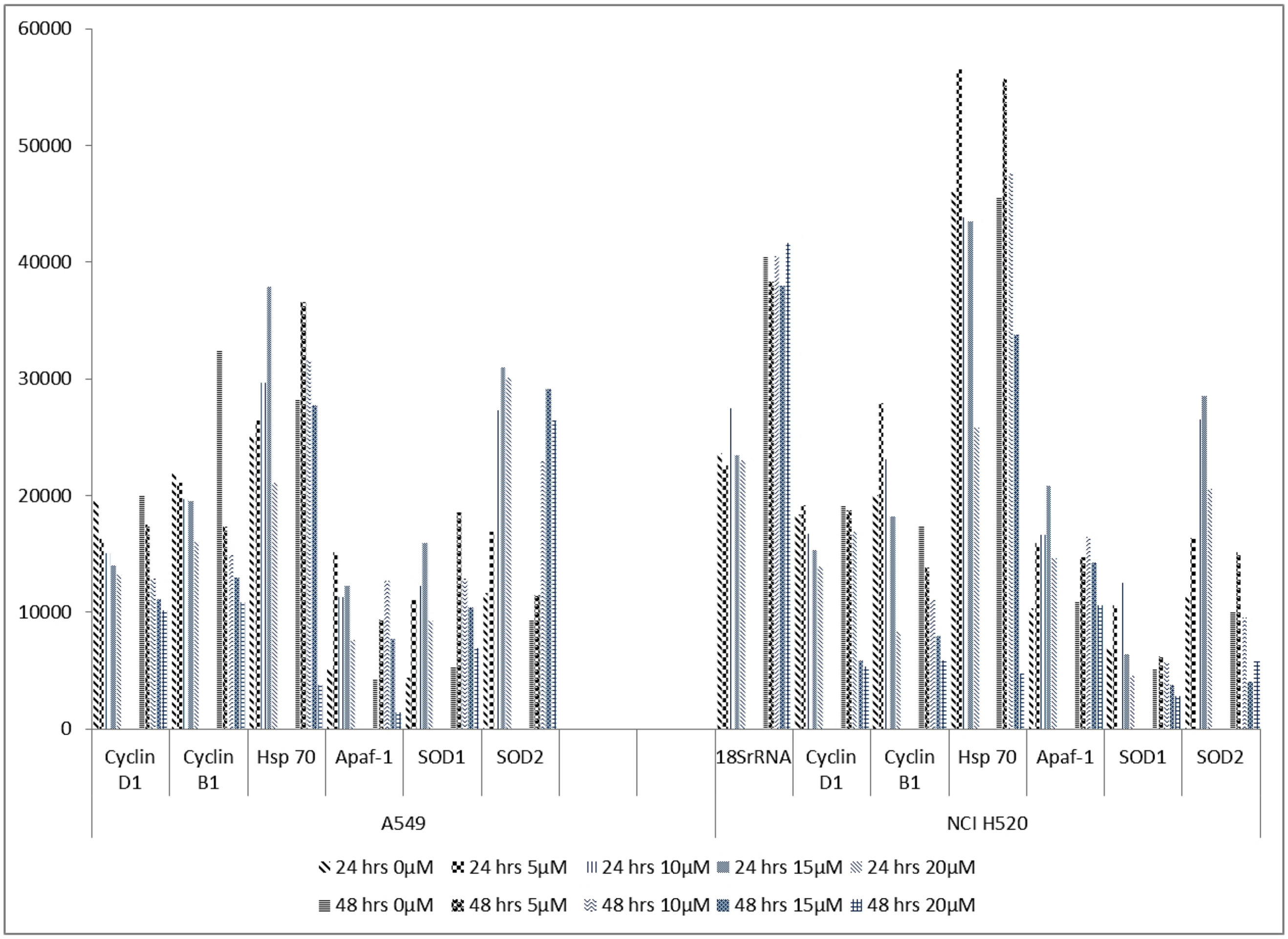

Supplementary Figure 3: Densitometric analysis of the mRNA level expression of genes studied by RT-PCR as shown in results.

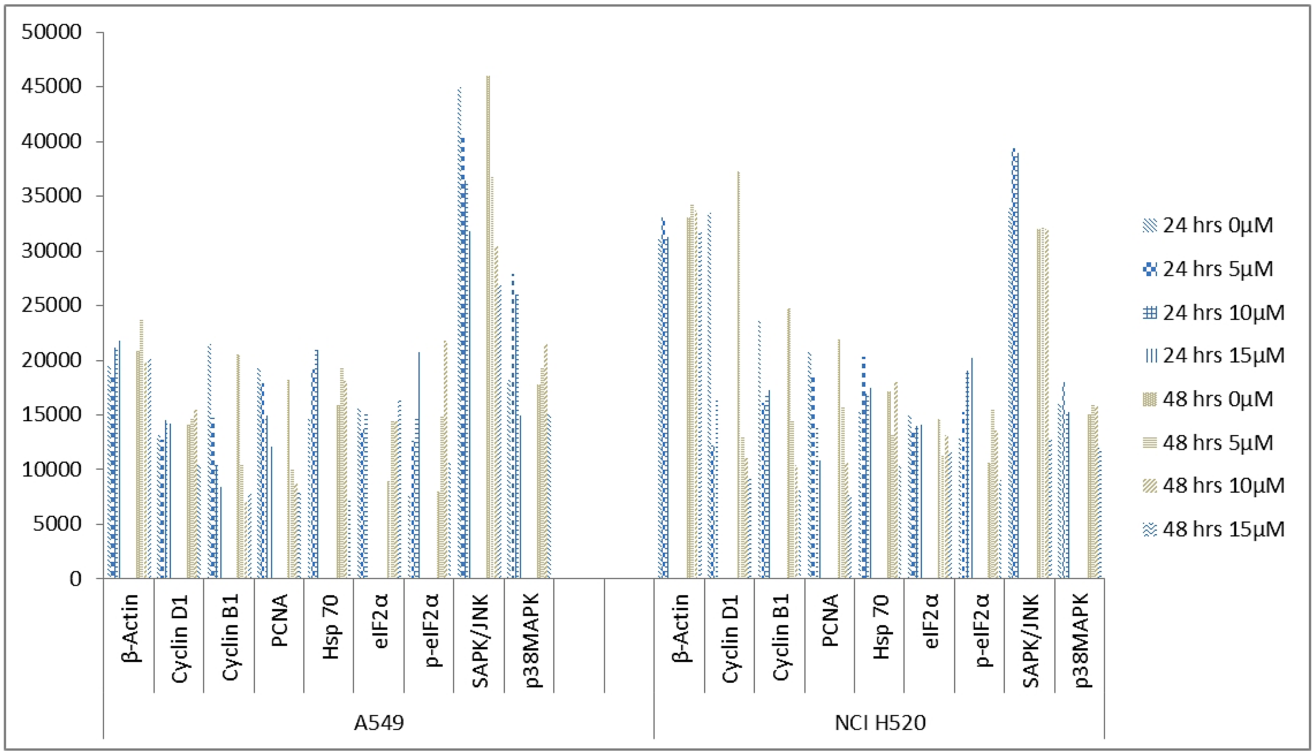

Supplementary Figure 4: Densitometric analysis of the protein level expression of genes studied by Western blot as shown in results.

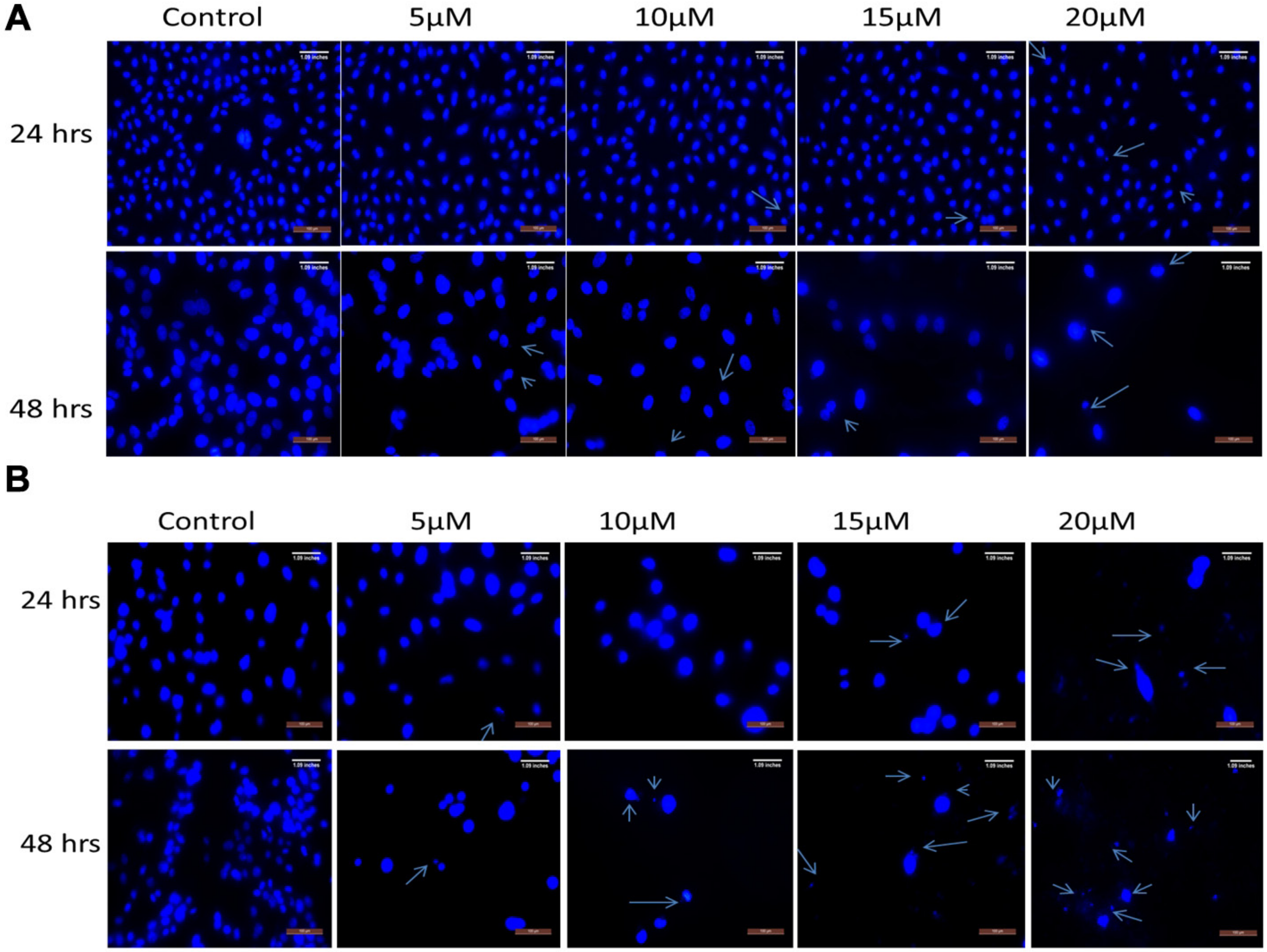

Supplementary Figure 5: (**A**) and (**B**) Nuclear staining of A549 and NCI H520 cells respectively using Hoechst 3342 for studying QC induced DNA damage and nuclear fragmentation. Cells were seeded in 6-well plates and exposed to various concentrations of QC. Post exposure cells were washed with PBS and stained with Hoechst 3342. Images were taken at 40× magnification (scale bar is 100 µM). The arrows represent fragments budding out from the genome.

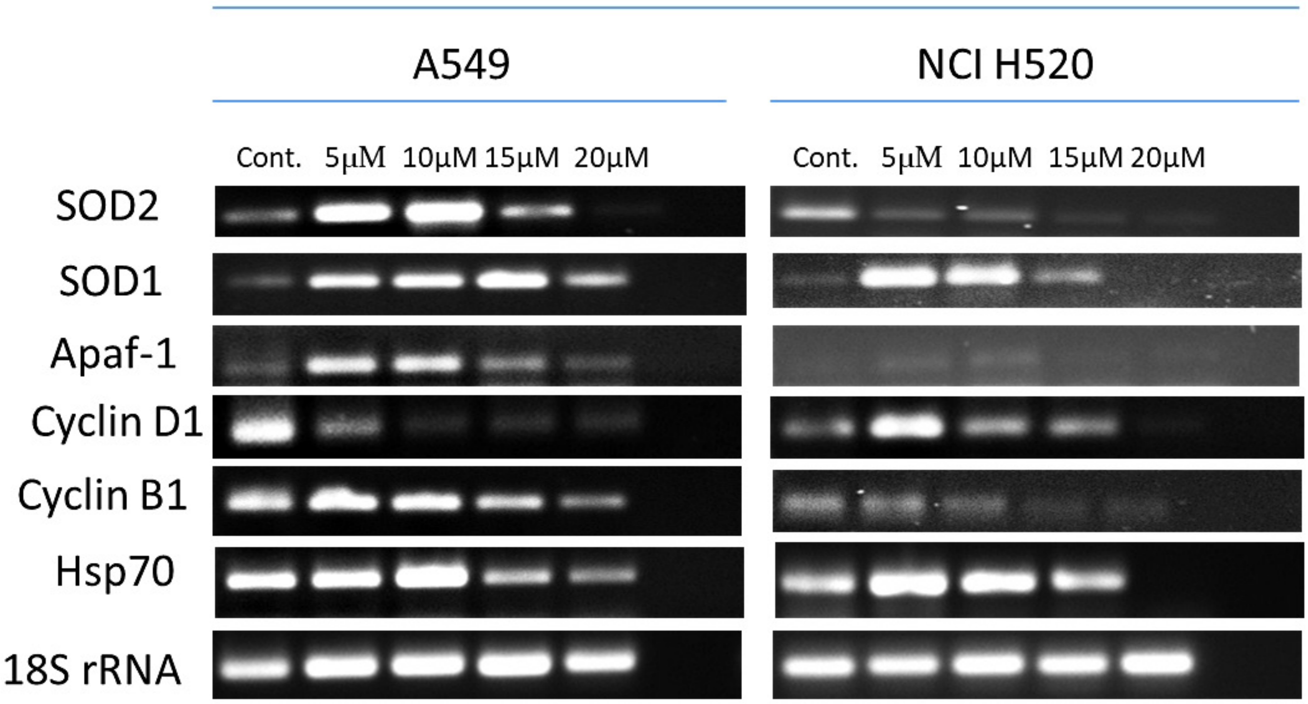

Supplementary Figure 6: Analysis of QC's effect on mRNA level expression of all genes as shown in results for 36 hrs time point.

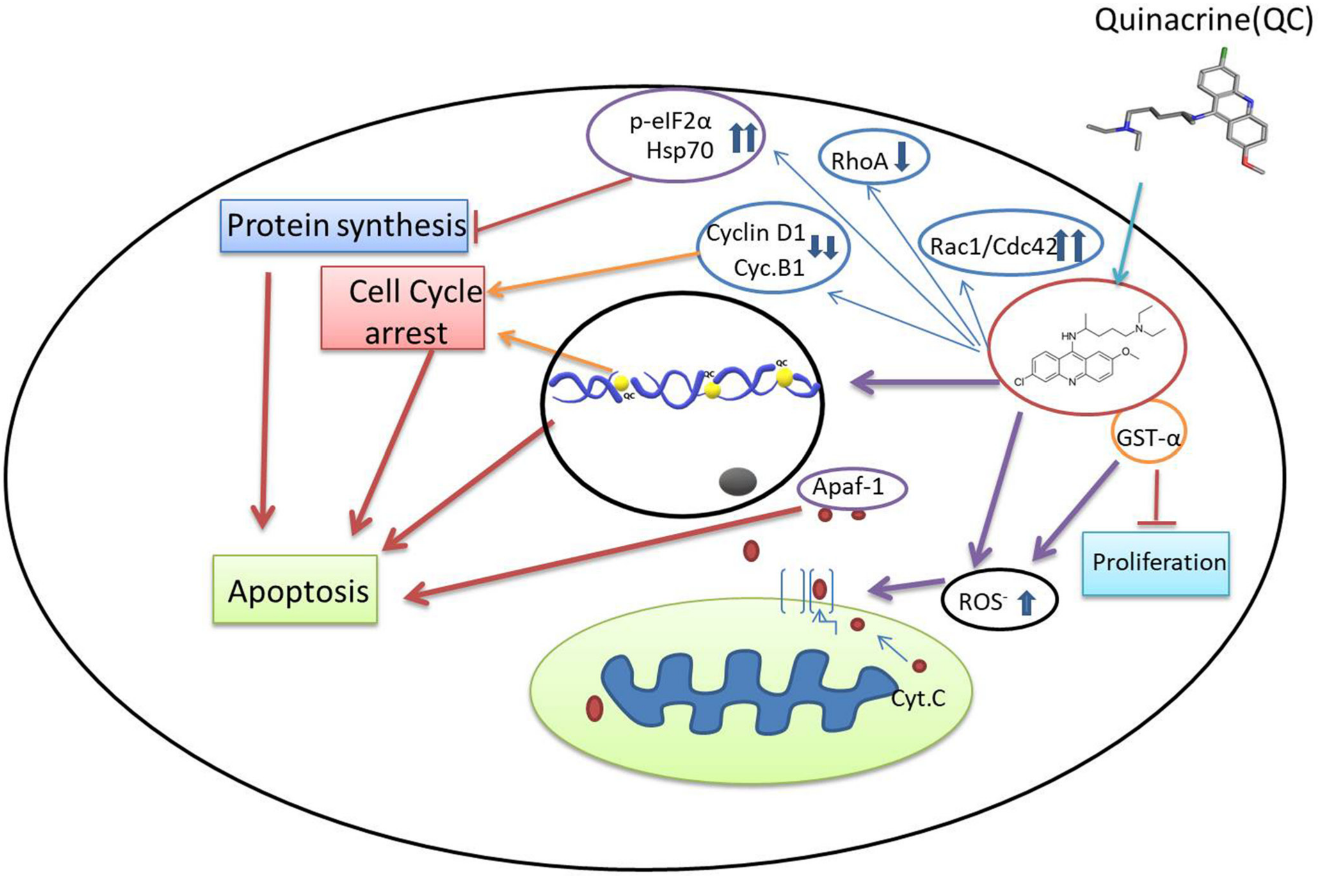

Supplementary Figure 7: Schematic representation of quinacrine's effect on NSCLC cells.
